# *The Wounded Lion* – Ageism and Masculinity in the Israeli Film Industry

**DOI:** 10.3389/fpsyg.2022.756472

**Published:** 2022-03-21

**Authors:** Shlomit Aharoni Lir, Liat Ayalon

**Affiliations:** The Louis and Gabi Weisfeld School of Social Work, Bar-Ilan University, Ramat Gan, Israel

**Keywords:** ageism, ageism and masculinity, aging stereotypes, aging discrimination, film industry, discrimination

## Abstract

One of the intriguing issues connected to power relations in the world of cinema that has yet to be adequately explored is what has happened over the years concerning the dominance and privilege of masculinity as signifying preferred social status. This qualitative study explores this subject based on transcribed semi-structured interviews with 13 award-winning Israeli directors over the age of 55. The research examines two questions: How has the film industry changed its relation to leading, award-winning film directors as they grow older? And, what challenges confront the directors, in terms of their own self-perceptions? These questions capture the collisional intersection between hegemonic masculinity and ageism, because they examine the loss of power attributed to men in the film industry as they reach the second half of their lives. The findings led to the formation of a theoretical model of ageism within the cinematic industry, allowing for the dismantling of the different factors that create obstacles for directors as they get older. The four layers of the model are: *arbitrary ageism*, manifested in the demands of a rapidly changing industry in a changing world; *passive ageism*, expressed through encounters with negative attitudes; *active ageism*, reflected in preventing older directors from receiving funding and from screening their films; and *self-ageism*, manifested in the directors’ attitudes toward themselves.

## Introduction

In old age, the social power derived from masculinity, as an emblem of centrality and privilege, is blended with social weakening, marginality and transparency that are associated in Western culture with advanced age. This phenomenon, which is referred to by Spector-Mersel (2006) as a paradoxical social category, can also be viewed as a *collisional intersection* that occurs when social signifiers of dominance are intermixed with those of perceived weakness.

The hegemonic and privileged place of Israeli men is interwoven into the fabric of Israeli society (Sasson-Levy, 2002). This is also apparent with regard to the world of Israeli films, which, from its starting days, was led by a majority of men directors and a minority of women directors (Shaer-Meoded, 2016). This significant male dominance was also reflected qualitatively, as male figures were the focus of the plots in most of the films (Shuv, 2002), a phenomenon that led to viewing the institutionalization of Israeli cinema as fundamentally masculine (Shohat, 1989). The Israeli masculine basis of the film industry reflects an international phenomenon: the birth of the film industry as gender-dependent stemmed from the accepted perception that masculinity and directing are interconnected, due to the technical aspects of the profession (Maher, 2001).

This masculine dominance in the world of film has led to ground-breaking research over recent decades, exploring the connection between gender and power relations in the world of film. As part of this effort, books, articles, and films have attempted to identify the reasons and the meaning of the very limited presence of women in the film industry, which has been dominated by men since its early days (Shaer-Meoded, 2016; Elefant et al., 2021; Aharoni Lir and Ayalon, 2022b). These studies pay attention to the question of male hegemonic power (Spector-Mersel, 2006) and discuss the significant minority of women directors, the phallocentric perspective of the cinema, and aspects of multiple marginalities, which mark subjugation as a product of the combination of a number of social systems (Miller, 2016). Nonetheless, one of the intriguing issues connected to power relations in the world of cinema, that has yet to be adequately explored, is what has happened over the years concerning the dominance and privilege of masculinity as signifying a preferred social status. This topic is especially interesting in view of the collision between age-based discrimination and status-based privilege.

In the current research, we explored the status of award-winning directors who hold the power to operate within their artistic world, involving crews and actors, as the long list of credits at the end of each film can indicate (Becker, 2008) and who might even be considered genius auteurs, for whom cinema is the pivotal center of being (Aharoni Lir and Ayalon, 2022a). Although behind the scene, film directors are the ones responsible for creating new worlds through motion pictures, and thus shape and affect public perceptions in extremely influential ways (John et al., 2017). The question that has yet to receive due research attention, is what happens to this immense power over time as the directors’ experience aging? This question is especially relevant, given widespread stereotypes that perceive creativity as belonging to youth (Simonton, 1990), alongside the common Western perception that the world belongs to the young (Heller and Hendeles, 1993).

This study explores what happened to the fame and respect that award-winning masculine Israeli directors enjoyed, after decades of work – based on two questions: How has the film industry changed its relation to leading, award-winning film directors, as they grow older? And what challenges confront the directors, in terms of their own self-perceptions?

### Masculinity, Film and Ageism

The field of masculinity studies developed several decades after women studies and was highly influenced by feminist theories. It released masculinity from transparent categorization that was viewed as presenting the norm (Connell, 2009). Early research in this field was divided between addressing masculinity as based on traits, and on viewing it as based on normative ideologies and seeing it as a socio-cultural product. However, contemporary theories conceive of masculinity as based on social norms which promise men privileges, with some men being more privileged than others (Thompson and Pleck, 1995; Spector-Mersel, 2006). Thus, while theories note the multiplicity of masculinities, and the inequality and the class tension, which exist between different forms of masculinity, they commonly acknowledge the existence of masculine hegemony that marks men in a collective way, as a privileged group, and promotes their status, in comparison to women in social structures (Connell and Messerchmidt, 2005; Connell, 2009; Dowd et al., 2012). This alludes to the fact that power is a dominant aspect in men’s experiences, actions, and social relations (Hammer, 1990). In Israel, hegemonic masculinity is perceived as a signifier of good citizenship. Thus, it plays a central role in the shaping of a gendered civic society based on a hierarchal order (Sasson-Levy, 2002).

This aspect is reflected in the film industry, which historically developed as a masculine industry in which male dominance has been maintained and directing has been perceived as being inherently masculine (Maher, 2001; Ward Mahar, 2001) and which continues to be perceived as such in many ways, to this day. In 2019, men directed 88% of the 100 biggest movies in the United States and 77% of the 250 leading movies during the same year. Eighty-six percent of the directors of the 500 leading movies in 2019 were male. Moreover, one-third of the films that were made in 2019 in the United States only employed men, as directors, writers, producers, editors and cameramen, or employed a single woman (Lauzen, 2012). This phenomenon is not unique to the United States; it is also common throughout the world (Aylett, 2016). As we describe below, the situation in the film industry in Israel is not different.

The average age of film directors in the United States has consistently increased over the years. On average, the age of directors of films released in 2000 was 45, whereas in 2019, it had risen to 50.6. Nonetheless, this industry is still largely considered a relatively young industry, with only 8.9% of directors who are over sixty (Stephen Follows, 2021; Film Data and Education).

In Israel, from the first days of the cinema, men dominated the field almost entirely and until the 2000s, only 7% of full-length films in Israel were directed by women. This gap has narrowed only in recent decades: between the years 2013 and 2018, women directed about one-fifth (21%) of the films released in Israel (Elefant et al., 2021). Data concerning the intersectionality of filmmaking and age in Israel are scarce and difficult to find. However, as this is a relatively young field in Israel, the early pioneers and directors who left their mark on the industry have now reached old age, which brings to the front their possible exposures to ageism.

Ageism is defined as a systematic bias in the way we think, feel, and behave toward people, because of their age. Ageism can be directed toward oneself, based on how one perceives the process of one’s own aging. However, ageism can also be directed toward others, for example, perceiving people as either too young or too old, to undertake activities or make decisions, solely because of their age. While ageism can be positive or negative, and directed toward any age group, research has focused mainly on older people and the negative social construction of old age. Ageism can occur at the institutional level via laws and regulations, but also at the interpersonal and individual levels (Ayalon and Tesch-Römer, 2017, 2018a).

Studies have found that older age is perceived as connected to a decline in standing in the social hierarchy (Robertson and Weiss, 2018). These attitudes are prevalent in varied fields, including the workplace, regardless of one’s physical state (Van der Horst and Vickerstaff, 2021). Ageism is also prevalent in interpersonal interactions. Interviews with older respondents have found that although the majority do not report explicit instances of ageism, they can identify interpersonal interactions in which they experience discrimination simply because of their age (Minichiello et al., 2000). Finally, these same negative age stereotypes that people grow up to accept as true of other older people, become self-relevant and self-fulfilling when they become older people (Levy, 2009).

In spite of the fact that research on ageism has increased over the last decade, the study of the intersection between ageism and sexism has only recently begun (Krekula et al., 2018). While older age marks women as less attractive, and hence, as having less feminine power (Krekula, 2007; Clarke, 2018), men are not perceived as losing their power as they age. Often the opposite is true: older age is associated with more social capital, experience, power and money, all of which connect to masculinity (England and McClintock, 2009; Shen, 2018). This is also reflected on screen, as older women have been excluded from the screen at a younger age, forced to take minor roles and their characters often reveal ageist and sexist stereotypes, older men have remained on the screen, openly portraying their physical change, even in their sixties and seventies (Markson and Taylor, 2000; Lauzen and Dozier, 2005). Less is known, however, about the situation behind the screen. Although the industry is highly masculine, the role of age and its impact on film directors’ careers have not been extensively explored.

Furthermore, it is only in recent decades that research has also begun to explore the losses that men face in old age (Spector-Mersel, 2006). The masculinity hegemony ranks young heterosexual men at the top and does not allow, and may even prevent, older men from being at the top (Calasanti, 2004). Hence, not all men share equal status and not all men are in power. In spite of the fact that the widespread stigma toward older men is less negative than the stigma toward older women, it is clear today that men are also exposed to negative stereotypes, because of their age (Roy and Ayalon, 2020). Moreover, a recent study has found that in the workforce, older men, rather than women, are more likely to experience ageism. This has been attributed to the norm, which expects older people to succeed and give the right of way to younger people. In contrast to men, women are seen as less threatening because of their reduced overall power in society. Thus, at older age, women actually manage to experience intersectional escape (Martin et al., 2019).

The question of Israeli award-winning directors is of particular interest in this regard, in view of their special status and wide acclaim. Although, in contrast to actors, film directors actually stand behind the camera and thus, are not necessarily in the public view, they still capture an extremely important role in shaping the public view and opinions as well as reflecting them. These people are considered extremely powerful, creating imaginary worlds and depicting real ones. In light of the important societal role of film directors, the study explores two questions: First, how has the film environment changed its relation to acclaimed directors over the years? Second, what challenges confront aging directors, in terms of their own self-perceptions?

## Materials and Methods

This qualitative study is based on an interpretative phenomenological analysis method (Smith and Osborn, 2015). We chose to conduct semi-structured interviews with 13 award-winning Israeli directors, who were 55 years or older at the time of interview. We chose award-winning directors with the thought it will help clarify the tension between the acknowledged and privileged position in the world of cinema and the cultural characteristics associated with old age.

We undertook a qualitative study to hear about the directors’ personal experiences concerning their age and social status as they describe them in their own words, reflecting their own perceptions. This method was especially important in view of the framework of lifespan time, proposed by Spector-Mersel (2006) to adopt a narrative perspective that views masculinity as a script that can develop and change with age and over time. The qualitative methodology made it possible to hear a range of voices from leading Israeli directors. Furthermore, it made it possible to discern common issues, by using an inductive, categorical approach, which relates to each interview as an individual marker, while also reaching an overall perception of the participants (Huberman and Miles, 2002). In this research, we decipher the aspects of age and gender from the viewpoint of the filmmakers, by focusing on the meaning of being a film director in a changing world.

Based on the criteria of age and notability in the film industry, we targeted possible participants and sent each director a personal email, asking him to participate in the study. Due to COVID-19, all the interviews took place via Zoom, over a few months, and were recorded and fully transcribed. The shortest interview lasted an hour and the longest, an hour and a half.

### Interview Guide

The semi-structured interviews were all conducted by the first author, starting with open and general questions about the choice of becoming a director and the relationship with the cinematic world. As can be seen in the Appendix, these were followed by more specific questions about changes in attitudes related to age and experience. The questions followed the lead of the directors and allowed them to openly respond to certain questions, refrain from answering others and make comments they found relevant. Not all questions in the interview guide were asked because the interviews were conducted in a free-flowing conversational mode. Following the response provided by the interviewee, new follow-up questions were introduced, and clarifications were sought when relevant. In almost all the interviews, the participants were at ease and spoke at length about their experiences, feelings, and insights from their many years of work in the film industry. A few of the directors avoided some of the questions and directed their talk to their areas of interest. One interview opened with the interviewee asking the interviewer questions in order to better understand whether he was indeed willing to participate in the study.

### The Analysis Process

The richness of the topics raised in the interviews, led us to divide the findings into several different articles. In the current research, we focused on the findings related most closely to the topic of age and generation in relation to the film industry.

The analysis consisted of the following stages. First, each interview was read several times and analyzed independently by focusing on the central themes it raised. This stage was conducted with no *a priori* coding scheme (Burnard, 1991). Instead, open coding was employed to reflect largely descriptive categories of meaning. For instance, in response to a question about the choice to direct films, one respondent stated: “It was a coincidence. I never dreamt of becoming a director.” A different respondent also provided a similar response: “It happened without my interference as I actually planned to write scripts.” Both statements were coded: an unplanned trajectory as a film director.

In the second stage, we noted the interpretive topics that were repeated in the interviews. For instance, the two examples above can be grouped into a broader category which characterizes film directing as a personal mission. This reflects a more interpretative level of analysis than the previous one. Another example is that in response to our queries about changes over time, we identified a repeated theme, which we framed and labeled as self-ageism. This is illustrated in the text below. In the third stage, we crosschecked the categories, which made it possible to discern common themes that corresponded with each other. Our analysis moved from the descriptive to the interpretive over time. To account for the rich data, we employed selective coding and focused our analysis on relevant themes to create a coherent storyline (Holton, 2007). Other themes that were deemed highly relevant to the present study concern the experiences of the film directors in the MeToo era and their ability to continue to create, despite their exposure to ageism. Although both themes are highly relevant to the findings discussed in this paper, we do not report these themes here, to remain as focused as possible on our main research questions. For further information about other products based on these interviews, please see Aharoni Lir and Ayalon (2022c,d).

### Trustworthiness

We employed several mechanisms to ensure the trustworthiness of the findings. First, our purposive sample addressed a diverse age group of film directors in their second half of life. Directors also differed regarding their sexual orientation and ethnic origin (their ethnicities included European, American, Arab and Middle Eastern countries). This diversity allows examining commonalities beyond differences in experience. We fully documented our analysis process to maintain an audit trail (Rodgers and Cowles, 1993). We provide thick descriptions to allow the readers to appreciate our thematic analysis (Ponterotto, 2006).

The study was approved by the Bar-Ilan University Ethics Committee. At the beginning of each interview, the directors were asked to give their consent to participate in the study after receiving an explanation that the interview would be transcribed and that their names would be used. Following substantial deliberation, we decided not to expose the directors’ names in academic manuscripts related to these interviews. This was done because the presentation of themes, rather than full interviews, might place some verbal statements out of context. As this is a very small community, we provide only limited information concerning the directors to maintain their confidentiality.

### The Research Sample

All the directors we targeted were well-known and highly acclaimed. The choice of age greater than 55 as a criterion was based on the fact that the perception of age is relative and people in different cultures associate different chronological ages with middle-age or old age (Ayalon et al., 2013). While the World Health Organization uses the age of 60 as a marker of old age, others relate to the 50 s as such a marker (MacLeod, 2015). Thus, the paper offers a wide range of aging experiences, starting with middle age or young old age, up to the old age group, with an average age of 71.

Table 1 presents the age of the directors and some of the prizes they have won.

**TABLE 1:** Directors by age and selected awards.

Name	Age	Selected award	Interview date
D1	73	The Film Arts Award; Venice Film Festival Award	17 July 2020
D2	55	Jerusalem Festival Award; Moscow International Film Festival Award	16 March 2020
D3	78	Lifetime Award; Ministry of Culture and Education Award	28 April 2020
D4	67	Silver Bear, The Ophir Award	13 May 2020
D5	71	Guggenheim Fellowship; Sundance Award	11 June 2020
D6	55	Washington Jewish Film Festival; Berlin International Film Festival	7 July 2020
D7	69	The Israeli Oscar; Atlanta Jewish Film Festival	10 June 2020
D8	68	Montréal World Film Festival; WorldFest Houston	12 April 2020
D9	79	The Israel Prize Award; Kinor David	1 April 2020
D10	64	Conrad Wolf Award; Berlin International Film Festival	2 July 2020
D11	84	The Israel Prize award; Zhytomyr Film Festival	3 May 2020
D12	78	The Ophir Award for Lifetime Achievements; International Film Festival of India	9 July 2020
D13	83	The Wolgin Award; Golden Bird Award	21 April 2020

#### Findings

It is important to note, that even though the group of directors was varied and included directors who faced obstacles and challenges, due to ethnicity and sexual orientation, this study focused on the stage in which the directors had already overcome these obstacles, as film directors, had won awards and gained acknowledgment in Israel and in the world. Therefore, these aspects are not reflected in this study.

In the analytical process we identified three major categories. The categories were: (a) *Changing World*: challenges related to changes that happened in the world of film; (b) *Societal Ageism*: the difficulties and pitfalls connected to ageism; and (c) *Self-ageism:* reflexivity and conceptualization of attitudes in relation to perceived changes associated with age.

### Changing World: “The World Is Changing and We Remain the Way We Are”

The interviews repeatedly described the challenges connected to the perception of age, time, and the professional changes that have occurred over the last few decades. The concept of living in a changing world was present in almost all the interviews and expressed in a wide range of aspects. One of these aspects, which unites the different descriptions, was the depth of the social change regarding the status of older adults and its connection to the world of the media:

In all the movies I made, the young and the old live in the same period and relate to the same world. When you talk about me and about my granddaughters… we love one another very much and talk to one another in a very open manner, but they already live in a different world.… when I see TikTok, for example, how much time they spend on this TikTok and what they do, and their short videos, that take me months to make, they make in a minute with simple programs on their I-Phones. They make fantastic short videos all the time. Now, that’s a different world (D13).

D13 notes that when he made movies in the past, young and old people had the same point of reference, and they shared the same world. However, today, not only do the young and old live in two different worlds, but, furthermore, closeness and love do not have the power to bridge these worlds. These understandings echo the separation that Prensky (2001) notes between the world before the information era and the one after it. Prensky compares older people to digital immigrants, who fear the new technological era and experience a significant gap between themselves and young people, whom he views as digital natives. This social separation is also consistent with the gerontological literature that argues that separation based on chronological age is a leading cause of ageism. Reportedly, if young and old people do not interact, they end-up forming two separate groups, rather than a single group in which group members care about each other (Hagestad and Uhlenberg, 2005).

Within this overall change, the directors also noted the significant change in the perception of cinema. This is what D11 said about the world of film that, in his opinion, has waned and lost its glory and significance:

French cinema flowed into me through the small movies, through the atmosphere of the time, everyone was so hot for the cinema. The cinema was so significant in the sixties. Later, the cinema began to dissipate. Once television, and of course, the new media began, the entire matter of film became less significant. So, I belong to this generation. I belong to the French New Wave. There, I was born as a filmmaker (D11).

D11’s words about the world of film that lost its grandeur reflect the manner in which new media technologies have changed the cinematic world (Mulvey, 2018). D11 places his point of reference and sense of origin, identification and belonging in a past in which the cinematic experience was wrapped in a sense of magnificence and glory.

The perception of a chasm between the cinematic experience of the past and that of the present is also reflected in D12’s words, as he notes the distinction between watching films in a theater and the experience of modern-day films, in which one sits in front of a small screen, full of advertisements:

There are many things that really bother me… you see a series and you are riveted and, suddenly, below appear words: “Teheran – at this and that hour” or “some sports star… was voted off the island.” And you say: I want to concentrate on this, and all the time there are all kinds of information. So, I say: I grew up on and developed by engaging in the films that I make for the big screen… there’s a movie that I am working on now, so I said: I don’t want it to be released on Zoom or streamed on the internet. I want the audience to sit in a movie theater and see it, so that they can laugh together and cry together… so I seem to be still connected to another period (D12).

This perception, lamenting a past in which watching a movie was a focused experience bound to the theatrical space, finds support in Mulvey’s (2018) ideas, which state that new technologies, such as video, DVD and the digital media, accelerate the death of the traditional cinema, by abolishing the place of the filmgoer as part of an audience. D12 presents the difference between the experience of the cinema, which makes it possible to concentrate on one movie, in a joint experience with an audience of people, and the modern and commercial world, in which films and series are often viewed on the computer or on television, which have commercials and other distracting information. This decreases the high status accorded to filmmaking in the past. From his point of view, the change abrogates the cinematic experience.

An expression of the totality of the change between the old cinematic world and the current reality can be found in the following description:

What did change is the number of creations and of creators. We watch an extensively larger number of movies, and visual expressions… so it might be that movies from the 60 s or 70 s stood out and excelled so centrally in new wave cinema – Bergman, Antonioni – they were prominent and controlled considerably the manner in which cinema developed. Today, the decentralization of various expressions because of technology, almost prevents the possibility of having one centrally compelling and controlling thing. So, I think that technological change is an expression of the changes that the world undergoes – the world has changed, we are changing …. We can’t, I can’t, maybe someone else can, really grasp the change, stop it, prevent it or run before it…. We can try and understand the change as much as possible and once we understand it, it has already changed. We are inside a rollercoaster that drives at an incredible speed, and we can at the outmost hold on strongly. We can never be before the change. In most cases we can fall, drift or get injured (D5).

It is quite apparent that D5 has given considerable thought to the topic of change, as he quite interestingly separates between two distinct periods of time: the past, which allowed gifted directors to become prominent figures in their field and lead it, and the present, in which this has become impossible. And while in his full words, D5 claims at length that the change is captivating and worth observing, the tone of his words can also indicate feelings of anxiety and fear due to the speed of change and inability to control it.

Overall, these various quotes reflect the directors’ mourning of a glorious past that is no longer available to them. They started out in an almighty industry, that celebrated the cinematic world. However, over time, their industry has lost its power and as discussed below, so have they.

### Societal Ageism: “Your Generation Has Already Done What It Had to Do”

Alongside descriptions lamenting a lost world and the changing industry of cinema, which caused a complete alternation in the frame of reference of their artistic trade, the directors shared experiences of changing societal attitudes toward them. The perception of the older filmmaker as someone, who is done creating and is no longer relevant in terms of filmmaking, was raised repeatedly in the interviews.

The older you get, you interest them less – as if there are already young and new good (directors), so you have this feeling, as if people are looking at you as if you are the thing that needs to be replaced…. so, often, if they get a script anonymously, they’ll read it; the thing itself still has relevance. So, it doesn’t matter if you’re over the age. *But, there’s the etiquette that that’s it. He’s already done it*. He’s made 25 films (D4).

D4 portrays the feeling that his amazing success of having made 25 films works against him instead of in his favor; he describes an alarming sense of having worked in an industry in which he excelled in the past, yet is now seen as someone that needs a replacement and has already passed his prime. This description of the attitude he faces stands in clear contrast to the passion with which he subsequently describes the creation of more movies based on new ideas about which he is tremendously passionate.

D13’s words demonstrate how this ageist attitude is not personal, but is stated, as an overall signifier, that relates to him as part of an entire generation:

What has happened now is that things change with age. Making films at my age is much harder. For television, for example, it’s not ‘‘sexy’’^1^. They want to connect to something “sexy.” That is, a guy can come and tell you: “Listen, *your generation has already done what it had to do. Enough, you don’t understand film*, television today” (D13).

This statement testifies to the expression of ageism and the ease with which young filmmakers devalue older directors in Israel. As a result, directors, who left their mark in the Israeli world of cinema, may find themselves in situations in which they lose the symbolic capital, respect, and prestige they had amassed over the years, because masculinity no longer grants them acknowledgment. Collective abolishment, of this sort, testifies to a reversal of the situation. In the past, masculinity was valued (Dowd et al., 2012), as was the positive reputation enjoyed by Israeli film directors, partially due to their belonging to the male social category (Shaer-Meoded, 2016). In the present, however, these older directors may find themselves in a disadvantaged state, due to their advancing age.

The sentiment, which is also felt by relatively young directors, is one of suspicion and being tested by agents and managers, who examine their talent under a microscope:

The world is really more suspicious about older people, about older directors… *there’s the question about how connected you are, how much you understand, how much you remain in the know* about changing things, what’s in fashion, about new cinema, can you do these things… there’s the fear, perhaps, that people say: “Perhaps we are investing in an old horse; perhaps, it’s worthwhile to find someone with whom we can begin to connect, upon whom to build great things, a person with strong shoulders, that’s a young guy. He’ll carry us on his shoulders” – be they agents or managers (D6).

This reaction corresponds with Van der Horst and Vickerstaff’s (2021) description of ageism in the labor market, which is based on the assumption that older people have less willingness to change. In the case of D6, who was only 55 at the time of the interview, the sense he expresses concerning the environment in which he works is that of suspicion and mistrust, which leads him to a position in which he has to “defend” or prove himself, despite his enormous success in the past. This description also accords with the feminist literature concerning politics, which affixes a judgmental and powerful gaze, as a praxis of oppression, against women (Kaplan, 1997). It is possible thus, to view relations that create otherness and non-acceptance as also present in the judgmental gaze that exists in the power relations between young and old men in the film industry. The older men experience suspicious, probing looks, and they feel that they are being controlled by men younger than them.

As agonizing as the ageist gaze is, what transpired as an even more difficult experience is the feeling of becoming transparent, especially after having reached great heights at a younger age:

There’s the thing from the beginning, yes, when they discover you and say. I can show you articles, or when they wrote about me in an Italian newspaper: “Early Fellini”… they really wrote things about me at the beginning, wonderful things. But, also afterward, later on, there were critiques. There’s this period when they write and you work and you continue, and then, suddenly, I think, the critics also say: “Wait a minute, there are new filmmakers, he’s not really special,” and so on… and then comes the period of apathy, yes, when it doesn’t matter what you do – you work really hard. I say: Look, in the last 10 years, I made three feature films… it doesn’t matter. *It’s as if you don’t exist…* and I hope that I’ll succeed – I don’t know, perhaps tomorrow… I’ll hang in there, and later they’ll say: “Okay,” “Not good,” “almost B+,” “not bad,” yes. They should come back and say that yes, something here is okay (D12).

What stands out in D12’s words, which describe how, when he was a young man, the world media compared him to the acclaimed Fellini, is not the negative reviews, but rather the feeling of being invisible, especially given the three movies he had made in the last 10 years. This finding expands existing research showing that not only women suffer from invisibility in old age (Ojala et al., 2016), though the grounds for making older men vs. women invisible in society might vary. It also adds a new layer in the understanding that well-acclaimed masculine figures do not necessarily escape this treatment, as it exists in critical reaction to their works. A relevant distinction is that of soft vs. hard ageism. Whereas soft ageism is manifested in interpersonal interactions in the workforce, hard ageism is the consequence of discriminatory laws and regulations (Stypinska and Turek, 2017). On the one hand, the approach of the film industry toward older film directors is seen as reflecting a case of soft ageism. However, on the other hand, given its pervasiveness in an almost organized fashion, it also reflects a case of institutional ageism, which is not dictated by law, yet obeys the unwritten laws of the industry.

Most prominently, ageism is also evident in the challenges the directors face when they wish to raise money from Israeli foundations to produce new films:

I can think of at least two directors from the older generation, both exemplary directors (names of the directors) who, let’s say, in the past 10–15 years have greater difficulty raising money…. There might be such a tone that the good ones already did what they had to do, now it is our time. It might be true about me too. I don’t know. I seldom think of the reasons for the refusals to support my films. I don’t see any good that comes from this (D10).

D10, a well-known director in his mid-sixties, started by talking about the difficulty of well-known directors in their 80 s and 90 s to raise money for their films. Then, he moved to talking about himself and his inability to raise funds for his movies in the last couple of years, which he had attributed to his advanced age.

A possible example of the experiences of film directors in the second half of life is provided by director D3:

I’m interested in the injury of the strong man. I’m interested *in the wounded lion*; it isn’t only that he’s wounded, but rather that *he needs to go deep into the forest so that others won’t see that he’s wounded*. The strong person’s injury reveals more layers and is more complex, if I make a hierarchy, which isn’t completely justified, but… the wound of the weak person is sometimes trivial – everyone will pity him, everyone will want to take care of him, etc., and it’s also clear. You need to discover the wound of the strong. That is, the crack in the man who is trying to be strong but can’t be either because he really is now hurt or something. He interests me because he is more complex, and he emanates many shades (D3).

This quote can be interpreted in reference to the experiences of the directors interviewed in this study. Indeed, they are privileged in many ways, yet, hurt underneath. The directors won numerous prestigious awards, inspired audiences, and controlled the cast and the cinematic scene for many years. Nevertheless, they also carry injuries and bruises, some of which they attributed to their experiences of ageism in the second half of life as part of the film industry.

### Self-Ageism: “I’ll Turn Into Something Else”

The interviews elicited reflexive processes of acknowledgment of what was happening, and different emotions that are connected to filmmaking at an older age. The directors conceptualized their emotions and pointed to the difficulty. However, they were also able to relate to these difficulties with humor. These different aspects reflect not only acceptance or adjustment, but also self-ageism, expressed by the internalization of the social norms that shape the perception of old age. Self-ageism may contribute to distancing from the group of people who are one’s age, due to identification with younger people and the social meanings connected to older age (Bodner, 2011). The sense of living in a world that has dramatically changed led some of the directors to express fear of being unable to communicate in today’s language and to look with a certain sadness at the strong foothold they had in the world of film in the past.

*I am very frightened*… I don’t know, but it seems to me that I am still speaking the language of the passing years… of the twentieth century. I haven’t succeeded in extricating myself from that stupid century. Unfortunately. But, that’s me (D3).

D3 openly expresses his sense of fear. He states that he feels that he belongs to a different time and age, which he tags as “that stupid century.” While he notes “that’s me,” which might show self-acceptance, the context of his words and his regret of not belonging to the current century point to the internalization of the past as a less valuable point of reference than the present.

A recent conceptualization of the experiences of older workers in the workforce relies on the GATE framework and takes into account: Generation (birth cohort), Age (life stage), Tenure (length of time in the organization), and Experience (skills accumulated over time) (North and Shakeri, 2019). This director acknowledges both his age and his cohort as limiting his ability to continue and direct in later life as the two are interconnected in his experiences.

Alongside this fear of losing the ability to be true to oneself, there were also signs of humor.

*I am a bit afraid* that I’ll turn into something else. That I’ll turn into something. Age is significant. But it seems to me that if you remain loyal and you don’t get stuck in some shtick of yourself and you don’t say “I’m a director like this and I do things like that” and you don’t speak about yourself in the third person… and you succeed in rummaging inside yourself and finding something new, each time you can carry on not too badly (D2).

Throughout his interview, D2, who was considered for years a young experimental director whose cinema has captured Israeli youth, the fear of aging was apparent. The adoption of negative age stereotypes, and the acceptance of these stereotypes as self-relevant represent a form of self-ageism (Levy, 2009). The problem with these self-directed stereotypes is that they become self-fulfilling when older people start behaving accordingly and may fail to fulfill their true potential due to fears that these stereotypes instigate (Ivan and Cutler, 2021).

The sense of ageism was also apparent in relation to exterior appearance. In the interview with D5, he related aging to loss: “If you had talked to me 10 years ago, you would have seen a blond director with blue eyes. Look what you have here today – a shadow of my former self” (D5).

This description is particularly interesting for two reasons: firstly, in view of the fact that directors are behind the camera, creators of a mini world interwoven of motion, vision and voice, it might be surprising that D5, who is highly established in his field, views himself in terms of his appearance in such negative terms. Secondly, gender wise, current literature suggests that women are more committed to visibility norms and care more about their appearance than men (Becker, 2008). The diminishing self-worth expressed by this director reveals that men are also not indifferent to the issue of appearance in old age. Whereas ageism is often attributed to women (Medina and Zecchi, 2020), men may also internalize Westernized discriminating perceptions of old age and aging (Heller and Hendeles, 1993).

## Discussion

In contrast to ageism experienced by women, which reflects intersectionality based on gender and age (Krekula, 2007; Clarke, 2018), the ageism the directors were exposed to, as it was revealed and discussed in our findings, reflects *collisional intersection*. This occurs when the gender-based status of masculinity provides social privileges, while advanced *age*-based status, leads to social weakening. The duality of this condition is best captured by the term “the wounded lion,” coined by one of the directors who took part in this study and is also reflected in the title of this manuscript. The term suggests that despite their many privileges and advantages, ageism serves as a bruising experience, that hampers the directors’ professional careers in later life. Thus, the metaphor of the wounded lion portrays the paradox between the social expectation from men to represent various aspects of the hegemonic ideal (Schrock and Schwalbe, 2009) and to construct their manhood by being “strong and silent” (Flurey et al., 2018) – and the reality of dealing with severe internal and external wounds that are associated with the second half of life.

Considering the social demands placed on men to appear strong and not reveal weakness (Real, 1999; Flurey et al., 2018), the interviews we conducted were surprising in the openness of some of the directors and their willingness to talk about their loss of social capital due to their older age. This openness issues a call to the Israeli film industry to acknowledge its bias, not only toward women, but also toward older directors and to take into account the concealed injuries of those who are considered socially strong, yet are subject to the experience of ageism.

When we studied the place of women in the Israeli film industry (Aharoni Lir and Ayalon, 2022b), we related to the fact that whereas there is an actual positive change with more women directors, they still must face *“celluloid hurdles*” manifested in the loss of precious time, due to the struggle with unique gendered obstacles. While we find it of extreme importance to study the disempowered position of women of all ages in the film industry, in this research we explored the positions of those who were marked as privileged. Our analysis has focused on the position of those who have been the strongest men in the Israeli film industry and delved into the “wound of the lion,” in which the injury has largely remained concealed.

By researching a privileged group that benefits from more opportunities, due to its gender and to the acclaimed status as well-known film directors, we were able to uncover aspects of ageism toward a strong social group of hegemonic masculinity. The findings demonstrate that men can experience aspects of social subjugation, similar to those found in research that describes women’s experiences (Shaer-Meoded, 2016).

### A Model of Ageism Toward Male Directors

The findings demonstrate the existence or experience of ageist attitudes that were also described by the relatively younger directors in the sample, who were in their late fifties and sixties. This is consistent with past research which has shown that it is the younger age groups that are particularly susceptible to ageism in the workforce (Chasteen et al., 2021). The findings allow for the formation of a model that consists of four layers of ageism in relation to the Israeli film industry: *arbitrary ageism*, *passive ageism, active ageism, and self-ageism*.

#### Arbitrary Ageism

Ageism is a social concept, which can be manifested in institutional norms and regulations, as well as in interpersonal interactions. We coined the term *arbitrary ageism* to indicate a phenomenon in which the vast changes in a specific industry can render prominent figures within the field less relevant and skillful as they age. While the decrease in status is not a personal matter and is caused by vast changes in numerous aspects related to the industry, in many cases it still triggers a sense of rejection and fear at the personal level.

#### Passive Ageism

This form of ageism is shown through a negative stance and disregard related to ageism. It was reported as manifested in various subtle, yet present ways; for instance, experiences of hostility and suspicion from people in the film industry, as well as being related to as one whose time has passed, with the person becoming invisible among people in the industry.

#### Active Ageism

Unlike *passive ageism*, which is indicated mainly by attitudes, which may cause a sense of inadequacy and invisibility, active ageism is a professional behavior that may sabotage filmmaking and films made by older directors. It was reported in experiences in which foundation directors and committees were unwilling to read manuscripts or invest in movies made by older directors and in the unwillingness to screen films made by older directors on traditional media platforms.

#### Self-Ageism

The manner in which some directors referred to themselves allowed us to also detect self-ageism, which was manifested in internalizing negative aspects of the self, based on age, and seeing oneself as less relevant and less capable.

These aspects of ageism demonstrate that the perception that older men maintain social capital (England and McClintock, 2009; Shen, 2018) does not accurately reflect reality, at least concerning Israeli directors. Like women, who become invisible at a certain age, because their physical appearance changes (Clarke, 2018), many directors also reported experiencing a lack of visibility and institutional discrimination, as they aged. While, in recent years, much attention has been given to the discrimination and deprivation experienced by women due to ageism (Bouson, 2016), research concerning ageism and men is still scarce. The issue of older male directors and ageism remains silent in the media, both in research and from the perspective of men themselves (Hazan, 2006). The present findings, however, help conceptualize the phenomenon and add to past research which has shown that older women are rated more positively than older men (Narayan, 2008).

When Millett (1969) described patriarchy, she wrote about a society that works according to two principles. The first is the control of women by men and the second is the control of younger men by older men. However, in our study, we arrive at a somewhat different conclusion. Regarding the film industry in Israel, the findings indicate the control of younger men over older men. This occurs both in terms of having the financial means and positions of power. A highly prominent, though somewhat contested explanation for the occurrence of ageism is modernization theory (Ayalon and Tesch-Römer, 2018b). Broadly speaking, the theory attributes the reduced status of older people to advancements in technology (Cowgill and Holmes, 1972). Consistent with this theory, the film directors, as well, identified significant technological advancements which have made some of their knowledge and skills irrelevant.

Having said that, it is important to note that almost all the directors we interviewed have found ways to continue to tell their story. Some engage in retelling their stories via somewhat different modes of communication, such as writing or making documentaries instead of feature films, while others continue to produce movies on a much tighter budget. A few have been able to continue as vigorously as they had before (Aharoni Lir and Ayalon, 2022b).

One good aftermath, which was discovered about a year and a half after concluding our interviews, was the realization that one major film foundation had allocated funding to support senior directors in spring 2022^2^. This much needed amendment gives hope for the possibility to correct the bias against the old.

Future research can examine these issues in other countries, thus allowing a wider understanding of the socio-cultural context of the phenomena. It can also examine the ability of masculine directors to continue their film craft despite the obstacles.

## Data Availability Statement

The dataset presented in this article are not readily available because request would be considered. Requests to access the datasets should be directed to the corresponding author.

## Ethics Statement

The studies involving human participants were reviewed and approved by the Bar-Ilan University Ethics Committee. The participants provided their written informed consent to participate in this study.

## Author Contributions

SA: concept development, initial draft, analysis, write-up, and critical revisions. LA: concept development, write-up, and critical revisions. Both authors contributed to the article and approved the submitted version.

## Conflict of Interest

The authors declare that the research was conducted in the absence of any commercial or financial relationships that could be construed as a potential conflict of interest.

## Publisher’s Note

All claims expressed in this article are solely those of the authors and do not necessarily represent those of their affiliated organizations, or those of the publisher, the editors and the reviewers. Any product that may be evaluated in this article, or claim that may be made by its manufacturer, is not guaranteed or endorsed by the publisher.

## Footnotes

1 In Hebrew, “sexy” is slang for innovative, out-of-the-ordinary.

2 As can be seen the Mifal Hapais foundation website on January, 2022 – https://my.pais.co.il/Pages/Landing.aspx?ProjectId=934 (Hebrew).

## Appendix

**Interview Guide**

**General questions**

Can you share with me what led you to the decision to become a film director?

What were your sources of influence on this decision?

In retrospect, what do you think about this decision? What are the pros and what are the cons?

**Questions about age and cinema**

In our study, we are interested in the ways in which masculinity is transforming over time. How do you perceive this topic in light of your experience? In cinema?

How do you perceive change over time in your cinematic approach?

What male protagonist represents masculinity in cinema for you? And why?

Can you name young protagonists that have been a source of inspiration for you?

Can you name old male protagonists that have been a source of inspiration for you? What was the source of the attractiveness?

How do you think Israeli men in cinema differ from men in American or European movies?

There is this argument that women disappear from the media once they reach a certain age. Does this also apply to men? Why? Why not?

How do you think age affects the representation of the protagonist in films?

**Specific questions about the creative world of the director**

What are some of the unique features of male protagonists in your cinematic world?

How do your protagonists cope with their aging process? Old age?

How have your male protagonists changed over time?

Has your approach to directing changed over time? If so, how?

How have sexual and gender issues changed in your films over time?

How are the films you produce now different from the ones you produced in the past?

**Questions unique to particular movies produced by the director**
